# Sparse Neighbor Joining: rapid phylogenetic inference using a sparse distance matrix

**DOI:** 10.1093/bioinformatics/btae701

**Published:** 2024-11-21

**Authors:** Semih Kurt, Alexandre Bouchard-Côté, Jens Lagergren

**Affiliations:** School of EECS and SciLifeLab, KTH Royal Institute of Technology, Stockholm, 100 44, Sweden; Department of Statistics, University of British Columbia, Vancouver, BC, V6T 1Z4, Canada; School of EECS and SciLifeLab, KTH Royal Institute of Technology, Stockholm, 100 44, Sweden

## Abstract

**Motivation:**

Phylogenetic reconstruction is a fundamental problem in computational biology. The Neighbor Joining (NJ) algorithm offers an efficient distance-based solution to this problem, which often serves as the foundation for more advanced statistical methods. Despite prior efforts to enhance the speed of NJ, the computation of the *n*^2^ entries of the distance matrix, where *n* is the number of phylogenetic tree leaves, continues to pose a limitation in scaling NJ to larger datasets.

**Results:**

In this work, we propose a new algorithm which does not require computing a dense distance matrix. Instead, it dynamically determines a sparse set of at most O(n log n) distance matrix entries to be computed in its basic version, and up to $O(n{\;\text{log}\;}^{2}n)$ entries in an enhanced version. We show by experiments that this approach reduces the execution time of NJ for large datasets, with a trade-off in accuracy.

**Availability and implementation:**

Sparse Neighbor Joining is implemented in Python and freely available at https://github.com/kurtsemih/SNJ.

## 1 Introduction

Inferring phylogenetic trees from molecular sequence data represents a core challenge in computational biology, and distance-based methods offer initial solutions, which often serve as foundational trees for more advanced statistical approaches (Price *et al.* 2010, Minh *et al.* 2020). Among these methods, the Neighbor Joining (NJ) algorithm stands out for its theoretical guarantees and relatively high accuracy. NJ is an iterative algorithm based on a distance matrix. In the context of a dataset with *n* taxa and *m* sites, computing the distance matrix requires $O(f(m)n^{2})$ time where f(·) is the function giving the complexity of alignment and distance computation per pair of taxa. Meanwhile, NJ’s iterative algorithm demands $O(n^{3})$ time, resulting in a total time complexity of $O(f(m)n^{2}+n^{3})$. This computational complexity presents a challenge in scaling NJ to large-sized datasets by modern standards.

Several efforts have been made to accelerate the canonical NJ algorithm, including Relaxed Neighbor Joining (RNJ) (Evans *et al.* 2006), Fast Neighbor Joining (FNJ) (Elias and Lagergren 2009), RapidNJ (Simonsen *et al.* 2008), NINJA (Wheeler 2009), and Heuristic Neighbor Joining (HNJ) (Clausen 2022). These techniques take the entire distance matrix as input and aim to reduce the $O(n^{3})$ time required for constructing a tree based on this matrix. RNJ achieves a practical runtime of $O(n^{2}\;\text{log}\;n)$ by relaxing the join criterion, though the worst-case complexity remains $O(n^{3})$. FNJ guarantees a time complexity of $O(n^{2})$ by avoiding exhaustive searches in each iteration and instead searching within a smaller set containing one pair per row in the distance matrix. Both RNJ and FNJ trade some accuracy for speed. RapidNJ and NINJA introduce an early stopping criterion by monitoring relations between the sorted and unsorted distance matrix, achieving practical runtimes of $O(n^{2}\;\text{log}\;n)$, although the worst case is still $O(n^{3})$. Employing a heuristic approach similar to FNJ and using dynamic equations, HNJ attains a worst-case runtime of $O(n^{2})$.

While the previously mentioned methods primarily focus on enhancing the iterative phase of NJ, which is asymptotically advantageous in the regime *m *<* n*, molecular sequence data, however, often exhibits a scenario where the number of sites is larger than the number of taxa (*m *>* n*) and much fewer previous work in the NJ literature has tackled the actual bottleneck encountered in these practical scenarios, that is the $O(f(m)n^{2})$ complexity of distance matrix computation (Clausen 2022). This complexity is $O(mn^{2})$ for linear-time computable distances on aligned sequences. However, it increases substantially when sequences are not aligned and/or distances are not linear-time computable. For example, if sequences require pairwise alignment, the computational cost of distance matrix computation can escalate to $O(m^{2}n^{2})$ (Russel 2014). Therefore, our focus in this paper is on addressing the distance matrix computation bottleneck associated with *m *>* n* scenarios, under the assumption that f(m)≥m. In this context, one can try to reduce either the number of distance computations or the cost of each distance computation. We have chosen to pursue the former approach. Specifically, we propose a statistically consistent and rapid distance-based algorithm, Sparse Neighbor Joining (SNJ), which does not require computing a dense distance matrix. Our approach relies on quartets to dynamically determine a sparse set of distance matrix entries to be computed. SNJ achieves a worst-case time complexity of O(f(m)n log n) in its basic version and $O(f(m)n{\;\text{log}\;}^{2}n)$ in an enhanced version, under the regime where *f*(*m*) > *n*. In addition to its favorable large-taxa scalability properties, SNJ can be used in the “online setting” where taxa are collected sequentially, in which case it is desirable to have approaches that avoid building the tree from scratch every time a new taxon is available (Fourment *et al.* 2018).

## 2 Related work

There is a rich literature on scaling phylogenetic inference methods to large number of taxa, see e.g. Zaharias and Warnow (2022) for a recent review. We focus this literature review on methods that use pairwise distance estimates as input—other methods such as likelihood-based methods have desirable properties such as statistical efficiency; however, there are situations where only pairwise branch length estimators are available, justifying continued interest in pairwise distance methods, for example, in complex evolutionary models where Felsenstein pruning is too costly, e.g. Bouchard-Côté (2013).

The computational complexity literature has thoroughly explored the problem of reconstructing phylogenies using a minimum number of pairwise distance queries (Hein 1989, Kannan *et al.* 1996, Kao *et al.* 1999). Both lower bounds and upper bounds are known to be O(n log n) (King *et al.* 2003). Most of these early papers did not consider the fact that pairwise distances are corrupted by noise, with some notable exceptions, e.g. King *et al.* (2003) considers noisy distance measurement arising from a Cavender–Farris evolutionary model. However, these methods were not benchmarked on datasets and we are not aware of publicly available software implementations.

Recently developed divide-and-conquer techniques for large-scale phylogeny estimation utilize disjoint tree mergers (DTMs) (Smirnov and Warnow 2020, Zaharias and Warnow 2022). This class of methods first partitions the species set into disjoint sets, which is generally performed by computing a starting tree via a fast method and decomposing this starting tree into multiple disjoint subsets of leaves (Molloy and Warnow 2019, Park *et al.* 2021). A tree on each subset is then constructed. Finally, a tree on the full dataset is pieced together from these subtrees using a DTM method. Our approach takes a distinct route, where the emphasis is on supporting the “online” setting, while Zaharias and Warnow (2022) and Smirnov and Warnow (2020) focuses on scalability via distributed computing.

FastTree (Price *et al.* 2010), a tool for approximately maximum-likelihood trees for large alignments, obtains its starting tree via a heuristic version of NJ that avoids computing a full distance matrix. By storing profiles of internal nodes instead of a distance matrix and using a collection of heuristics, FastTree’s heuristic NJ achieves $O(mn\sqrt{n}\;\text{log}\;n)$ complexity for aligned sequences. However, it lacks the theoretical guarantees provided by our method (see Section 3.3). Additionally, it requires a multiple sequence alignment (MSA), limiting its potential use cases compared to our method (such as serving as a guide-tree for MSA methods, see Section 4).

Outside of phylogenetics, other methods have been developed to rapidly construct trees from pairwise data. For example, MSA software typically constructs a guide-tree (Sievers and Higgins 2017) used to iteratively align groups of sequences. However, these methods lack the consistency guarantees provided by our method (Section 3.3).

A concurrently and independently developed method, dnctree (Arvestad 2023), uses a top-down approach to address the aforementioned bottleneck, partially avoiding full distance matrix computation and achieving an empirical complexity of O(f(m)n log n) under certain tree distributions, but with a worst-case scenario of $O(f(m)n^{2})$.

## 3 Materials and methods

### 3.1 Background

We start by reviewing the NJ algorithm. NJ first requires the pairwise distance matrix *D* to be computed. For a dataset of *n* taxa and *m* sites, the time complexity of this step is $O(f(m)n^{2})$. NJ proceeds to compute the following for each taxon *i*:

$$u_{i}=\sum_{j:j\neq i}^{n}D_{ij}/(n-2).$$

Next, it joins the pair *i*, *j* for which $D_{ij}-u_{i}-u_{j}$ is the smallest. Then, it updates the distance matrix as follows:

$$D_{(ij),k}=(D_{ik}+D_{jk}-D_{ij})/2.$$

NJ keeps joining taxa until they form a single taxon. The time complexity of this entire joining process is $O(n^{3})$. Together with the distance matrix computation, the total complexity becomes $O(f(m)n^{2}+n^{3})$. It is possible to reduce it to $O(f(m)n^{2}+n^{2})$ using one of the aforementioned optimizations of NJ. For the regime *f*(*m*) > *n*, however, the $O(f(m)n^{2})$ complexity of distance matrix computation is the bottleneck in scaling NJ to large datasets.

### 3.2 Sparse Neighbor Joining

We propose a statistically consistent method that eliminates the need for a dense distance matrix and thus scales better for large data. The method begins with a small tree and incrementally updates it by inserting one taxon at a time.

In the following, *T^k^* denotes the topology of an unrooted, bifurcating tree with *k* leaves $l_{1},l_{2},\ldots ,l_{k}$. Although branch lengths are computed when placing additional leaves on a given tree, they are not stored in our iterative algorithm. If the user requires branch lengths, they can be obtained via optimization in a post-processing step.

#### 3.2.1 Updating a backbone tree with a new taxon

Let $l_{k+1}$ denote a new taxon to be placed on the “backbone” tree *T^k^* to form a new tree $T^{k+1}$. Placing this single new leaf $l_{k+1}$ is equivalent to picking one of the 2k−3 edges in the backbone tree *T^k^*. We propose an algorithm capable of making this edge selection in a logarithmic number of steps. The proposed algorithm is based on the following lemma:

Lemma 1. *Given a tree T with k leaves, there exists an internal node whose removal partitions the tree into connected components, each with at most* k/2*leaves (Jordan 1869).*

We refer to such a partitioning node as the centroid of the tree. Given a backbone tree *T^k^*, the algorithm initially decomposes the tree into three subtrees by removing the centroid (but not any edges). It then carefully picks one of these subtrees and calls itself recursively until the selected subtree consists of only one edge. Note that the number of recursive calls is bounded by the logarithm of the number of nodes in *T^k^*.

Given a subtree *S*, the careful selection of the next subtree *S_next_* is done as follows. First, the algorithm finds the centroid *c_S_* of the given subtree *S*. The removal of *c_S_* from *S* induces three subtrees: $S_{a},S_{b},S_{c}$. For each subtree, the algorithm randomly picks a leaf and designates it as an orienting leaf for that subtree. Using the new taxon $l_{k+1}$ together with three orienting leaves $\left\{l_{a},l_{b},l_{c}\right\}$, it constructs a quartet *Q* via NJ. Based on *Q*, we can determine which subtree $l_{k+1}$ should be inserted into. For example, if Q is $\left[l_{a},l_{k+1}|l_{b},l_{c}\right]$, then *S_next_* is selected as *S_a_*.

In cases where one or more of the subtrees $S_{a},S_{b},S_{c}$ lacks leaves, the partition of *T^k^* by the removal of *c_S_* is considered: $T_{a}^{k},T_{b}^{k},T_{c}^{k}$. Given that $T_{i}^{k}$ covers *S_i_* for i∈{a,b,c}, the algorithm picks a leaf *l_i_* from $T_{i}^{k}$ and assigns it as an orienting leaf for *S_i_*. Using these orienting leaves, it applies the same strategy as described above to decide *S_next_*.

#### 3.2.2 Full phylogenetic topology inference

The idea proposed in the previous section can be turned into full phylogenetic topology inference. For a given set of *n* observed leaves $L=\{l_{1},l_{2},\ldots ,l_{n}\}$, we can randomly select 4 out of them and construct a quartet. Then, we can treat this initial quartet as the backbone tree (*T*^4^) and insert the rest of the leaves one by one using the process presented in the previous section. This proposed algorithm is referred to as Basic SNJ and summarized in Algorithm 1. For each insertion, the number of quartets to be constructed is bounded by O(log n) and the number of pairwise distance computations needed per quartet is constant. As a result, the worst-case time complexity of the proposed method is O(f(m)n log n), excluding the cost associated with finding centroids.

An additional computational cost, which turns out to be negligible in our *f*(*m*) > *n* setup, is incurred when determining centroids. Even with a naive implementation based on enumeration, the centroid for a tree can be found in linear time. Given that the size of the subtree is reduced by half at each step, the time complexity of identifying all the centroids required for an insertion remains linear (O(n+n/2+…+1)=O(n)). Consequently, the overall additional time complexity is bounded by $O(n^{2})$. Critically, this part of the time complexity does not scale in *m*. So while more efficient centroid update algorithms may be possible, we do not focus on this issue since in practice (and in theory in the regime $f(m)n\;\text{log}\;n>n^{2}$), the centroid computation cost is negligible.

Algorithm 1. Basic SNJ algorithm

1: Randomly select 4 leaves from the set of leaves *L*

2: Remove the selected leaves from *L*

3: Construct the initial phylogeny *T*^4^

4: k←4

5: **for** *l* in L **do**

6:  $S\leftarrow T^{k}$

7:  **while**|S|≠1**do**

8:   Get centroid *c_S_* and subtrees $S_{a},S_{b},S_{c}$

9:   Sample orienting leaves $l_{a},l_{b},l_{c}$ from $S_{a},S_{b},S_{c}$

10:   Construct a quartet Q using $\left\{l,l_{a},l_{b},l_{c}\right\}$

11:   Select $S_{\mathrm{next}}\in \{S_{a},S_{b},S_{c}\}$ based on Q

12:   $S\leftarrow S_{\mathrm{next}}$

13:  **end while**

14:  Get $T^{k+1}$ placing *l* on the edge *S*

15:  k←k+1

16: **end for**

The performance of the proposed method can be improved by picking orienting leaves based on a heuristic criterion instead of uniformly at random. We investigate one heuristic criterion which involves choosing leaves that are as close as possible to the new taxon. However, since computing the pairwise distance between the new taxon and every leaf in a subtree is computationally expensive, we propose an approach where we first sample log n leaves from each subtree and then select the one closest to the new taxon as the orienting leaf. Due to the distance computation between log n sampled leaves and the new taxon, the worst-case time complexity of the method becomes $O(f(m)n{\;\text{log}\;}^{2}n)$.

The performance of the proposed method can be further boosted without increasing its worst-case time complexity. One way of doing it is by picking more than one orienting leaf from each subtree and averaging the resulting distances before forming a quartet. This strategy improves the probability of quartets having the correct topology while multiplying the total number of pairwise distance computations only by a small constant. Another way to improve performance is to construct the initial backbone tree with more than four leaves. This approach enables the algorithm to use more than one orienting leaf even for the initial insertions. For instance, $k_{0}=\sqrt{n\;\text{log}\;n}$ leaves can be initially used without exceeding the previously calculated complexity. This choice requires n log n pairwise distance computations for the formation of the initial tree, thus remaining consistent with the previously established worst-case time complexity while enhancing the overall performance.

The SNJ algorithm, incorporating all of the aforementioned improvements, is outlined in Algorithm 2.

Algorithm 2. SNJ algorithm

1: Randomly select $k_{0}=\sqrt{n\;\text{log}\;n}$ leaves from the set of leaves *L*

2: Remove the selected leaves from L

3: Construct the initial phylogeny $T^{k_{0}}$

4: $k\leftarrow k_{0}$

5: **for** *l* in L **do**

6:  $S\leftarrow T^{k}$

7:  **while**|S|≠1**do**

8:   Get centroid *c_S_* and subtrees $S_{a},S_{b},S_{c}$

9:   Sample log n leaves from each of $S_{a},S_{b},S_{c}$

10:   Pick the closest 3 orienting leaves per subtree:$\begin{array}{c} L_{a}^{\mathrm{ort}}=\{l_{a}^{1},l_{a}^{2},l_{a}^{3}\},\;\\ L_{b}^{\mathrm{ort}}=\{l_{b}^{1},l_{b}^{2},l_{b}^{3}\},\\ \;L_{c}^{\mathrm{ort}}=\{l_{c}^{1},l_{c}^{2},l_{c}^{3}\} \end{array}$

11:   Construct a quartet Q using average pairwise distances of $l,L_{a}^{\mathrm{ort}},L_{b}^{\mathrm{ort}},L_{c}^{\mathrm{ort}}$

12:   Select $S_{\mathrm{next}}\in \{S_{a},S_{b},S_{c}\}$ based on Q

13:   $S\leftarrow S_{\mathrm{next}}$

14:  **end while**

15:  Get $T^{k+1}$ by placing *l* on the edge *S*

16:  k←k+1

17: **end for**

### 3.3 Theoretical guarantees

#### 3.3.1 Convergence radius

We will use the following proposition for the convergence radius theorem of SNJ:

Proposition 1. *Let* T*be the true bifurcating tree for the leaf set* $L=\{l_{1},l_{2},\ldots ,l_{n}\},\;\lambda (\cdot )$*be a function that gives the shortest branch length in a given tree, and D be the distance matrix computed using L. Given D, NJ returns the true tree* T*if:*

$$\underset{ij}{\max}|T_{ij}-D_{ij}|<\frac{\lambda (T)}{2},$$

*where* $T_{ij}$*denotes the sum of branch lengths in the shortest path between i and j in the true tree.*

Proof. See Atteson (1999) and Elias and Lagergren (2009).

Since SNJ employs NJ to construct the initial tree and quartets, it is possible to prove the same convergence radius for SNJ.

Theorem 1. *Given D, SNJ returns the true tree* T*if:*

$$\underset{ij}{\max}|T_{ij}-D_{ij}|<\frac{\lambda (T)}{2}.$$

Proof. By induction on the iteration *k*. We first prove the result for the initial tree *T^k^*, which is obtained using NJ on the subset $L^{k}=\{l_{1},l_{2},\ldots ,l_{k}\}$, *k* = *k*_0_. Let *D^k^* be the distance matrix for the *k* leaves in *L^k^*. Let $T^{k}$ denote the tree obtained by removing the leaves not in *L^k^* from the true tree T. The shortest branch length in $T^{k}$, given by $\lambda (T^{k})$, is greater than or equal to λ(T). The largest error in *D^k^*, on the other hand, is less than or equal to the largest error in *D*, i.e.

$$\underset{ij}{\max}|T_{ij}^{k}-D_{ij}^{k}|\leq \underset{ij}{\max}|T_{ij}-D_{ij}|<\frac{\lambda (T)}{2}\leq \frac{\lambda (T^{k})}{2}.$$

Therefore, following from Proposition 1, *T^k^* will have the same topology as $T^{k}$.

The next leaf, $l_{k+1}$, will be placed via recursive subtree selections according to quartets constructed by NJ. For any quartet *Q*, NJ considers 4 leaves. Let *L*^4^ denote any subset of leaves with size equal to 4 and *D*^4^ be the corresponding distance matrix. Using the same argument as above with *k *=* *4:

$$\underset{ij}{\max}|T_{ij}^{4}-D_{ij}^{4}|\leq \underset{ij}{\max}|T_{ij}-D_{ij}|<\frac{\lambda (T)}{2}\leq \frac{\lambda (T^{4})}{2}.$$

Therefore, following from Proposition 1, it is guaranteed that any quartet *Q* will have the correct topology. That means $l_{k+1}$ will choose the right subtree in each recursive call and finally will be placed on the correct edge, making $T^{k+1}$ have the true topology.□

#### 3.3.2 Statistical consistency

In this section, to make the manuscript self-contained, we present a simple union-based argument establishing the statistical consistency of SNJ.

For simplicity, we consider in our experiments a Jukes–Cantor model of sequence evolution, which admits the following closed-form branch length maximum likelihood estimator (Felsenstein 2004):

$$D_{ij}^{\left(m\right)}={\hat{T}}_{ij}=-\frac{3}{4}\text{log}(1-\frac{4}{3}\frac{m_{ij}^{d}}{m}),$$

where $m_{ij}^{d}$ is the number of different sites between taxa *i* and *j*, and *m* is the total number of sites. The present analysis could be straightforwardly generalized to other evolutionary models.

We start with a lemma establishing that for each pair of taxa *i*, *j*, the branch length estimator $D_{ij}^{\left(m\right)}$ converges in probability to $T_{ij}$, the sum of branch lengths in the shortest path between *i* and *j* in the true tree.

Lemma 2. *For any fixed i, j, as m goes to infinity*, $D_{ij}^{\left(m\right)}$*converges to* $T_{ij}$*in probability, i.e.*

$$\forall \epsilon >0,\underset{m\to \infty }{\lim}P(|D_{ij}^{\left(m\right)}-T_{ij}|>\epsilon )=0.$$

Proof. Follows from standard consistency results on maximum likelihood estimators (van der Vaart 2000).

Next, using a simple union bound, we can guarantee that *all* entries in the distance matrix are eventually within a fixed error bound with high probability.

Lemma 3. *As m goes to infinity*, ∀ϵ>0*, the probability of any entry in the full distance matrix* $D^{\left(m\right)}$*having an error greater than ϵ converges to zero:*

$$\forall \epsilon >0,\underset{m\to \infty }{\lim}P(\underset{ij}{\max}|D_{ij}^{\left(m\right)}-T_{ij}|>\epsilon )=0.$$

Proof. Let $A_{ij}^{\left(m\right)}$ be the event that the *ij* element of $D^{\left(m\right)}$ has an error greater than *ϵ*: $|D_{ij}^{\left(m\right)}-T_{ij}|>\epsilon$. Then, the event that at least one element has a greater error than *ϵ* is $\underset{ij}{\cup }A_{ij}^{\left(m\right)}$, where i,j∈[1,2,…,n]. From Boole’s inequality:

$$P(\underset{ij}{\cup }A_{ij}^{\left(m\right)})\leq \sum_{ij}P(A_{ij}^{\left(m\right)})\;\;.$$

Applying Lemma 2:

$$\begin{array}{l} \underset{m\to \infty }{\lim}P(\underset{ij}{\cup }A_{ij}^{\left(m\right)})\leq \underset{m\to \infty }{\lim}\sum_{ij}P(A_{ij}^{\left(m\right)})\\ =\sum_{ij}\underset{m\to \infty }{\lim}(A_{ij}^{\left(m\right)})\\ =0. \end{array}$$

□

Using the above two lemmas and Theorem 1, we can now state and prove the consistency of SNJ.

Theorem 2. *As m goes to infinity, the SNJ estimator converges with probability 1 to the true tree* T:

$$\underset{m\to \infty }{\lim}P(T_{m}=T)=1,$$

*where T_m_ denotes the tree estimated from SNJ based on distances computed with m sites.*

Proof. Lemma 3 states that as *m* goes to infinity, the largest error in $D^{\left(m\right)}$ will be less than *ϵ*, ∀ϵ>0. Setting *ϵ* as $\frac{\lambda (T)}{2}$ implies that as *m* goes to infinity, *D* ends up in the convergence radius of SNJ, guaranteeing the reconstruction of the true tree T according to Theorem 1.□

### 3.4 Evaluation metrics for accuracy

A classical metric used to compare phylogenetic trees is the Robinson–Foulds (RF) distance (Robinson and Foulds 1981). Previous work has shown that the RF distance is rapidly saturating, leading to imprecision (Lin *et al.* 2012): the RF distance considers partitions of taxa implied by edges, and computes the proportion of the partitions existing in both trees, therefore, RF does not take into account how similar or different two partitions are, rather, it only checks if they are identical.

As distance-based methods offer preliminary solutions that can be refined by more complex statistical approaches, the need arises for a sophisticated distance metric capable of recognizing similarity between edges that are similar but not identical. The transfer distance (TD) (Day 1981, Charon *et al.* 2006, Lin *et al.* 2012) is such a metric suitable for comparing an inferred tree against a reference tree (Lemoine *et al.* 2018). For each reference edge, TD identifies the closest edge (i.e. the closest partition) in the inferred tree, calculates the number of taxa required to move across this edge to match the reference edge, and then normalizes this number by the size of the smaller partition side implied by the reference edge. The average of these values across all edges yields the transfer distance for the entire tree.

We also report the quartet distance as it is another metric that does not saturate easily, making it suitable for comparing trees that are not highly similar (Steel and Penny 1993). The quartet distance is based on the proportion of quartets that differ between two trees (Sand *et al.* 2014).

## 4 Results and discussion

**Figure 1.**
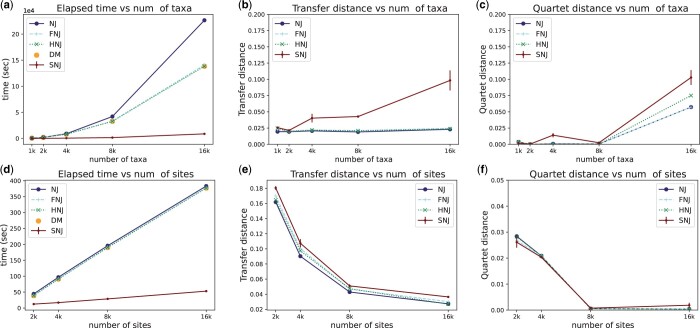
(a–c) Runtime and accuracy results for different numbers of taxa, where the number of sites is 20k. (d–f) Runtime and accuracy results for different numbers of sites, where the number of taxa is 1k. SNJ results are averaged over different seeds. Error bars show standard error. Note the scale multiplier in the time plots: for example, the computational time in the 16k taxa regime goes from 138 392 s (about 40 h) to <8700 s (about 2.5 h).

We first demonstrate the time efficiency of SNJ on synthetic data. Synthetic data were generated in two steps: sampling a topology and simulating sequences. A random topology is sampled as follows. Each leaf is initially treated as an independent taxon. Then, a pair of taxa are picked at random and merged into one taxon. This random pairing and merging process continues until a single connected component is obtained. Every branch length is then drawn from an exponential distribution with the scale parameter of 1e−3. Having a topology sampled, sequences are simulated using the Jukes–Cantor model of sequence evolution. By this way, we simulated five different datasets where the number of taxa, *n*, varied from 1000 to 16 000, while the number of sites, *m*, was fixed at 20 000. On these datasets, we compared our python implementation of SNJ with NJ through scikit-bio (scikit-bio development team 2020), FNJ through fastphylo (Khan *et al.* 2013), and HNJ through CCPhylo (Clausen 2022). We chose FNJ and HNJ over other alternatives because they represent the current fastest optimizations of NJ, offering guaranteed quadratic running times. The elapsed wallclock time during inferences were measured and presented in Fig. 1a. Runtime for each method includes the computation of pairwise Hamming distances it needs. We also separately show the time for computing the upper triangulars of the complete distance matrices (denoted as “DM”), to give a better depiction of the aforementioned bottleneck. As clearly seen from the results, SNJ scales remarkably better with the increased number of taxa. The results also reveal that this improvement in scalability cannot be achieved by any method that requires a full distance matrix. The accuracies of the inferred trees were assessed by comparing them against the true topologies using the transfer (Fig. 1b), quartet (Fig. 1c), and RF (Supplementary Material S1) distances. While SNJ achieved comparable transfer distances to the other methods for the initial two datasets, there were some accuracy compromises for the subsequent datasets, as the sparsity of distance matrix entries used by SNJ increases with the number of taxa. However, even for these datasets, the SNJ trees remained close to the true tree, as indicated by the transfer distance score being <0.1. The increase in accuracy loss can be attributed to the fixed sequence length of 20 000. With a larger sequence length, SNJ may maintain pace with the other methods in terms of accuracy for larger numbers of taxa. While the results for RF distances confirm the accuracy gap, in terms of quartet distance SNJ followed the other methods more closely.

Following the aforementioned data generation process, we simulated additional datasets keeping the number of taxa, *n*, at 1000 and varying the number of sites, *m*, from 2000 to 16 000. Subsequently, the previously mentioned runtime and accuracy experiments were conducted for this data (Fig. 1d–f and Supplementary Material S1). The results indicate that the previous optimizations FNJ and HNJ are not effective in *m *>* n* setting, because they do not address the bottleneck caused by distance matrix computation. On the other hand, SNJ scales notably better with the increased number of sites. Moreover, it achieved comparable accuracies with the other methods, and all methods yielded better accuracies with larger numbers of sites.

We performed further experiments on synthetic data to investigate the effects of the numbers of initial leaves, sampled leaves, and orienting leaves on the accuracy and runtime of SNJ. See Supplementary Material S2 for details.

**Figure 2.**
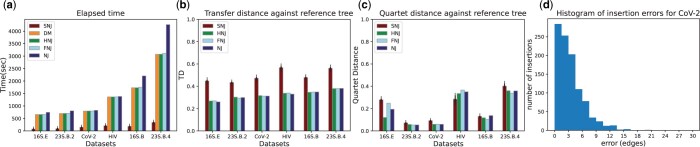
(a) Elapsed time and (b and c) accuracy results on six different empirical datasets. SNJ results are averaged over different seeds. Error bars show standard error. (d) Histogram of individual insertion errors for the CoV-2 dataset.

The time efficiency and accuracy of SNJ were further evaluated on six empirical datasets. Table 1 provides information regarding both the quantity (numbers) and size (lengths) of aligned sequences in the mentioned datasets. Figure 2a presents the runtime results, including the computation time of pairwise Hamming distances. As evident from the outcomes, SNJ exhibited a noteworthy speed advantage, being faster on all the datasets and scaling better with the number of taxa. For accuracy analyses, a reference tree for each dataset was generated by running IQ-TREE-2 (Minh *et al.* 2020) employing 100 parsimony trees + BIONJ (Gascuel 1997) tree as starting trees for tree search. The trees produced by NJ, FNJ, HNJ, and SNJ were then compared against these reference trees using the transfer (Fig. 2b), quartet (Fig. 2c), and RF (Supplementary Material S3) distances. Our method achieved comparable quartet distance results with the other methods, giving the best result for HIV data. On the other hand, in terms of transfer and RF distances, it traded-off some accuracy but showed a stable performance over different datasets.

**Table 1.** Summary of datasets.

Dataset	Taxa	Seq. length	Type	Reference
16S.E	1938	7465	rRNA	Cannone *et al*. (2002)
23S.B.2	2024	7668	rRNA	Cannone *et al*. (2002)
CoV-2	1123	27 669	RNA	Hadfield *et al*. (2018)
HIV	1275	34 603	RNA	Abecasis *et al*. (2007)
16S.B	3067	7774	rRNA	Cannone *et al*. (2002)
23S.B.4	4050	7668	rRNA	Cannone *et al*. (2002)

To obtain a deeper insight into the comparison between canonical NJ and SNJ, we conducted an additional experiment on the CoV-2 dataset where we analyzed the performance of each taxon insertion individually. For each leaf, we calculated the error as the number of edges between the edge chosen by SNJ and the edge to which the leaf belongs in the NJ tree. To prevent errors from propagating through subsequent insertions, we adjusted the leaf’s position after calculating the individual distance, moving it to the same location it occupies in the NJ tree. This experiment was repeated five times, and the results are depicted in a histogram in Fig. 2d. The histogram reveals that the vast majority of insertions exhibit quite small deviations compared to the NJ tree. In fact, more than half of them fall within a three-edge vicinity of where NJ would place them. These findings suggest that if SNJ insertions are followed by a local correction method that focuses solely on the vicinity of the insertion and thus has low complexity, the trade-off in accuracy can potentially be mitigated, keeping the speed advantage.

The scalability of SNJ was further assessed on a larger dataset, KmerFinder (Clausen *et al.* 2018, Sayers *et al.* 2019). KmerFinder provides the complete distance matrix for 23 331 complete bacterial genomes. Since not the sequences but the distances are available for this data and the distance matrix computation would be dominating the runtime otherwise, we consider the number of pairwise distances utilized by each algorithm as the computational cost. NJ, FNJ, and HNJ require all the pairwise distances, which is $\frac{23 331\cdot 23 330}{2}\approx 272M$. SNJ, on the other hand, used only ∼4.7M pairwise distances to build a tree on 23 331 taxa. This number corresponds to 0.0175 of the total number of pairwise distances, indicating that the time efficiency is expected to become even more evident for huge datasets exceeding several hundred thousand taxa/sites. Since the sequences were not available for this data, we could not run IQ-TREE-2 to obtain a reference tree, therefore an accuracy analysis was not feasible.

**Figure 3.**
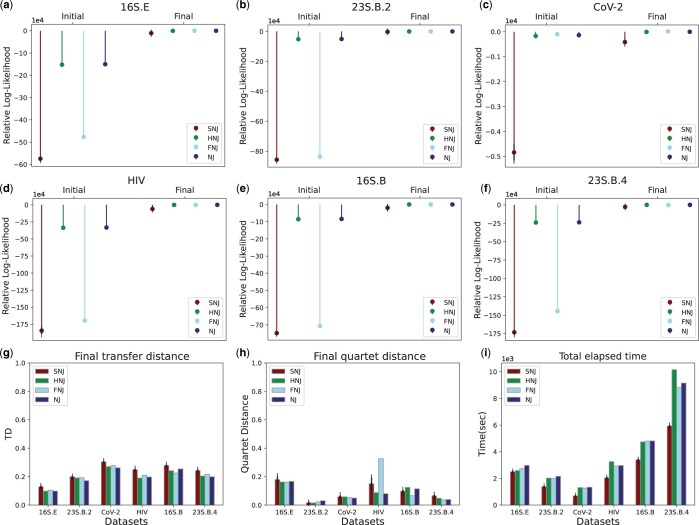
(a–f) Initial and final relative log-likelihood results of IQ-TREE-2 initiated with trees generated by NJ, FNJ, HNJ, and SNJ. (g) Transfer distances between the final and reference trees. (h) Quartet distances between the final and reference trees. (i) Total [starting tree construction + nearest neighbor interchange (NNI) operations] elapsed time results. SNJ results are averaged over different seeds. Error bars show standard error. Note the scale multipliers in the time and log-likelihood plots.

To provide another way of accuracy assessment and show experimental evidence for the previous claim that small accuracy loss in the starting tree of an advanced statistical method will not appreciably affect the final result, we conducted the following experiment on the six empirical datasets. We started IQ-TREE-2 with trees produced by NJ, FNJ, HNJ, and SNJ, and examined the initial log-likelihoods and the log-likelihoods after 100 iterations. Instead of absolute log-likelihoods, we report log-likelihoods relative to the log-likelihoods of the previously obtained reference trees, as done in De Maio *et al.* (2023). Figure 3a–f displays the initial and final relative log-likelihood results. The initial log-likelihoods of SNJ and FNJ were lower compared to those of HNJ and NJ. After 100 iterations, however, the differences among the log-likelihoods of different methods are substantially reduced. We further evaluated the accuracies of the final trees using the transfer (Fig. 3g), quartet (Fig. 3h), and RF (Supplementary Material S4) distances. As seen from the results, the final trees for all methods exhibited comparable distance scores, showing once more that SNJ’s trade-off in accuracy is rapidly mitigated by subsequent nearest neighbor interchange (NNI) operations. For time analysis, we measured the corresponding total (starting tree construction + NNI operations) elapsed times. As Fig. 3i demonstrates, starting IQ-TREE-2 with SNJ proved to be the fastest method for all datasets, saving more than 50 min for the largest dataset, 23S.B.4. Overall, this experiment indicates that advanced statistical methods can be initialized with SNJ to gain a speed advantage without substantial sacrifice in the accuracy of a final tree.

The potential use of SNJ in phylogenetics extends beyond advanced statistical methods. SNJ can also be applied in the divide-and-conquer pipelines of DTMs, which typically require either a starting tree or a guide-tree to partition the set of leaves into multiple disjoint subsets (Park *et al.* 2021, Zaharias and Warnow 2022). For example, Molloy and Warnow (2019) utilizes NJ for this purpose. Replacing NJ with SNJ for a speed advantage is likely to maintain the effectiveness of the leaf partitions, as SNJ accurately inserts most leaves into their correct vicinity. Thus, SNJ can accelerate the partitioning step of divide-and-conquer pipelines without compromising their overall performance.

The applications that can benefit from SNJ are not limited to phylogenetic inference. MSA methods typically construct a guide-tree from pairwise data and use it to iteratively align groups of sequences. While current MSA methods can use NJ to build their guide-trees, the complexity of NJ limits its use to small numbers of sequences. For larger datasets, they resort to other methods or heuristics to rapidly construct trees. For example, Edgar (2004) uses UPGMA and Sievers and Higgins (2017) employs a bisecting k-means approach, both of which are not statistically consistent. Thus, there is a need for a scalable and statistically consistent method that can work with pairwise aligned data in MSA. Given SNJ’s speed, consistency guarantees, and ability to handle pairwise aligned data, it has significant potential to fulfill this need.

## 5 Conclusion

In this work, we presented a distance-based rapid phylogeny inference technique. The proposed method, SNJ, directly addresses the current bottleneck in scaling NJ to larger datasets. This bottleneck arises from the need to compute the *n*^2^ entries of the distance matrix, where *n* is the number of phylogenetic tree leaves. Unlike previous distance-based methods, SNJ does not require computing a dense distance matrix; instead, it dynamically determines a sparse set of at most $O(n{\;\text{log}\;}^{2}n)$ distance matrix entries to be computed. As a result, SNJ offers improved scalability for large datasets.

SNJ has been proven to be statistically consistent. However, it prioritizes speed over accuracy, leading to some degree of accuracy loss. We demonstrated that this loss of accuracy is primarily due to the accumulation of minor individual insertion errors, which can be quickly corrected by a subsequent statistical method, making SNJ a good candidate for a rapid starting tree in advanced statistical methods. Additionally, since SNJ accurately places taxa in their correct vicinity, it can be employed in the divide-and-conquer pipelines of DTMs to rapidly partition the species set. Furthermore, given its scalability and consistency guarantees, SNJ holds significant potential for use in MSA methods to construct guide-trees.

One possible future work is equipping SNJ with a local statistical correction method. The basic idea is that once SNJ finds the correct vicinity for an insertion, this local correction method can be used to refine the placement with greater precision. This would lead to a hybrid phylogeny inference method that combines distance-based and statistical approaches.

## Availability and implementation

*Source code*: Available at https://github.com/kurtsemih/SNJ.

*Sequence data*: 16S.E, 16S.B, and 23S.B.2-23S.B.4 data are retrieved from Comparative RNA Web Site and Project (CRW): https://crw-site.chemistry.gatech.edu/DAT/3C/Alignment/Files/16S/16S.E.ALL.alnfasta.zip, https://crw-site.chemistry.gatech.edu/DAT/3C/Alignment/Files/16S/16S.B.ALL.alnfasta.zip, https://crw-site.chemistry.gatech.edu/DAT/3C/Alignment/Files/23S/23S.B.ALL.alnfasta.zip. CoV-2 data is retrieved from Nextstrain: https://data.nextstrain.org/files/ncov/open/global/aligned.fasta.xz. HIV data are retrieved from the HIV Sequence Database: https://www.hiv.lanl.gov/components/sequence/HIV/search/search.html.

After following the above link, select “complete genome” for the “genomic region” field during the search. In the download options, choose “align” and mark “Pad aligned sequences.” Then, you will receive a download link for the HIV data.

Sequences with excessive gaps were omitted from all datasets during the experiments. To obtain the exact subsets used, one should select the sequences with the fewest gaps (see Table 1 for the number of selected sequences in each dataset).

*Distance data*: KmerFinder data are retrieved from the platform ScienceData operated by the Technical University of Denmark: https://sciencedata.dk/shared/8da6465076fa9e75197a4ccf1b2b7d07?download.

## Supplementary Material

btae701_Supplementary_Data
