# Supplementary material for: Sparse Neighbor Joining: rapid phylogenetic inference using a sparse distance matrix
Source: Bioinformatics. 2024 Nov 21;40(12):btae701. doi: 10.1093/bioinformatics/btae701 (PMC11637600; doi:10.1093/bioinformatics/btae701)
Supplement: btae701_Supplementary_Data [file btae701_supplementary_data.pdf]

Supplementary for Sparse Neighbor Joining: rapid phylogenetic inference using a sparse distance matrix

1 RF distance results for synthetic data

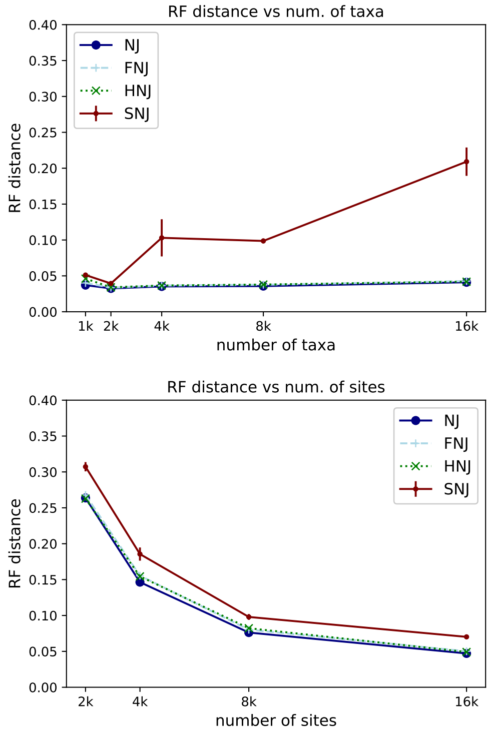

Supp. Fig. 1: RF distance results for synthetic data: (Top) RF distance vs. number of taxa and (Bottom) RF distance vs. number of sites.

2 The effects of SNJ parameters

SNJ utilizes a configuration of $\sqrt{n \log n}$ initial leaves, $\log n$ sampled leaves, and 3 orienting leaves. Initial leaves are used to construct a backbone tree, sampled leaves are a randomly sampled subset of leaves at a subtree, and orienting leaves are a subset of the sampled leaves that are closest to the new taxon. To explore the impact of each parameter, we ran SNJ with varying numbers of initial leaves, sampled leaves, and orienting leaves on the synthetic data with 1 000 taxa and 4 000 sites. Throughout our investigation, we maintained the other two parameters at their default values to isolate the effects of the parameter under inspection. Experiments were repeated with different seeds. Supp. Fig. 2 presents the results regarding transfer distance against true tree and runtime. As expected, increases in these parameters led to higher accuracies but also longer runtimes. Notably, a diminishing return phenomenon emerged concerning accuracy as the numbers of sampled or orienting leaves increased (Supp. Fig. 2A,C), whereas runtimes exhibited a linear increment (Supp. Fig. 2B,D). This trend supports our choice of 3 orienting leaves and $\log n$ ($= 9$, in this case) sampled leaves, since SNJ prioritizes speed over accuracy. The increase in the number of initial leaves, on the other hand, exhibited a more gradual, linear improvement in accuracy (Supp. Fig. 2E). However, its impact on runtime is expected to escalate faster than linearly (Supp. Fig. 2F), discouraging the adoption of larger numbers. Therefore, the default setting of $\sqrt{n \log n}$ ($= 99$, in this case) strikes a balance by achieving a modest enhancement in accuracy without any significant increase in runtime.

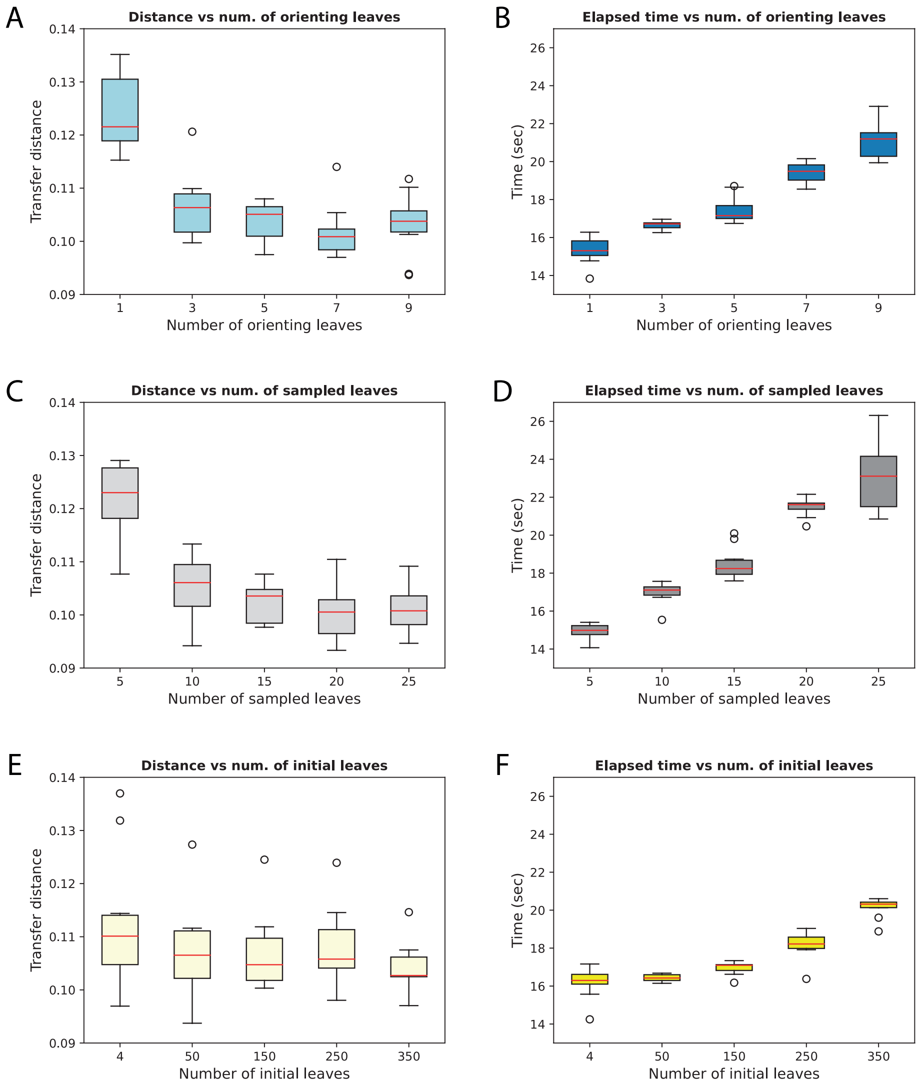

Supp. Fig. 2: The impact of varying numbers of (A-B) orienting leaves, (C-D) sampled leaves, and (E-F) initial leaves, on accuracy and runtime.

3 RF distance results for empirical data

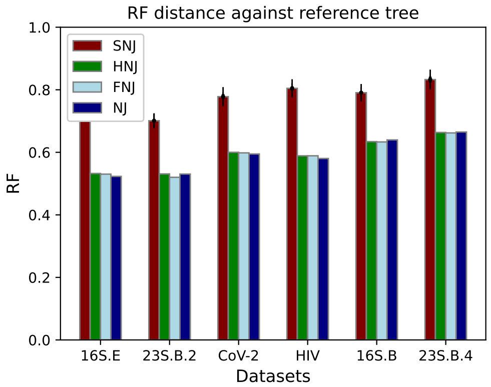

Supp. Fig. 3: RF distance results for six different empirical datasets.

4 RF distance results for final IQ-TREE-2 trees

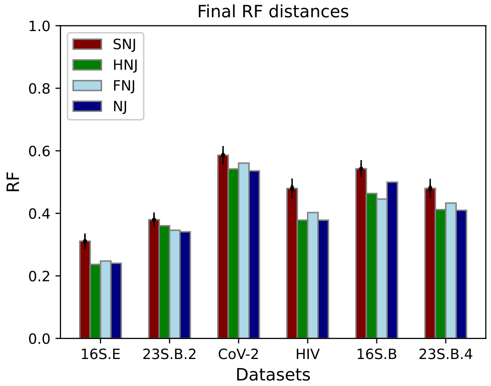

Supp. Fig. 4: RF distances between the final and reference trees, when IQ-TREE-2 is initiated with NJ, FNJ, HNJ, and SNJ.
